# Controlling Superselectivity of Multivalent Interactions with Cofactors and Competitors

**DOI:** 10.1021/jacs.2c06942

**Published:** 2022-09-14

**Authors:** Tine Curk, Galina V. Dubacheva, Alain R. Brisson, Ralf P. Richter

**Affiliations:** † Department of Materials Science and Engineering, Northwestern University, Evanston, Illinois 60208, United States; ‡ Département de Chimie Moléculaire, Université Grenoble Alpes, CNRS UMR 5250, 38000 Grenoble, France; § UMR-CBMN, CNRS-University of Bordeaux-IPB, 33600 Pessac, France; ∥ School of Biomedical Sciences, Faculty of Biological Sciences, School of Physics and Astronomy, Faculty of Engineering and Physical Sciences, Astbury Centre for Structural Molecular Biology, and Bragg Centre for Materials Research, University of Leeds, Leeds, LS2 9JT, United Kingdom

## Abstract

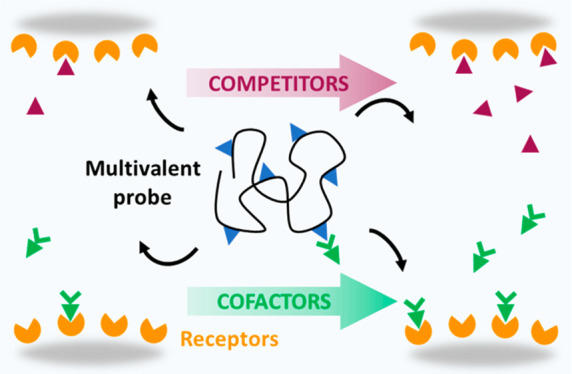

Moieties that compete with multivalent interactions or act as cofactors are common in living systems, but their effect on multivalent binding remains poorly understood. We derive a theoretical model that shows how the superselectivity of multivalent interactions is modulated by the presence of cofactors or competitors. We find that the role of these participating moieties can be fully captured by a simple rescaling of the affinity constant of the individual ligand–receptor bonds. Theoretical predictions are supported by experimental data of the membrane repair protein annexin A5 binding to anionic lipid membranes in the presence of Ca^2+^ cofactors and of the extracellular matrix polysaccharide hyaluronan (HA) binding to CD44 cell surface receptors in the presence of HA oligosaccharide competitors. The obtained findings should facilitate understanding of multivalent recognition in biological systems and open new routes for fine-tuning the selectivity of multivalent nanoprobes in medicinal chemistry.

Multivalent interactions involve the simultaneous formation of multiple supramolecular bonds, such as ligand–receptor binding^1^ or host–guest complexation.^2,3^ The combinatorial entropy of possible binding configurations gives rise to a supralinear change in the number of bound multivalent probes as a function of receptor concentration.^4,5^ This superselective behavior^6^ allows specific targeting of surfaces displaying binding sites above a threshold surface concentration, while leaving surfaces with lower coverages virtually unaffected (Figure 1A). The types of multivalent entities that display superselectivity vary widely, including proteins,^7^ antibodies,^4,8^ polymers,^9,10^ viruses,^11−13^ liposomes, and nanoparticles.^14,15^ Resolving the mechanism of multivalent interactions is crucial both to understand the selectivity of biomolecular interactions and to facilitate the design of highly selective nanoprobes for diagnostics and therapies.^16^

**Figure 1:**
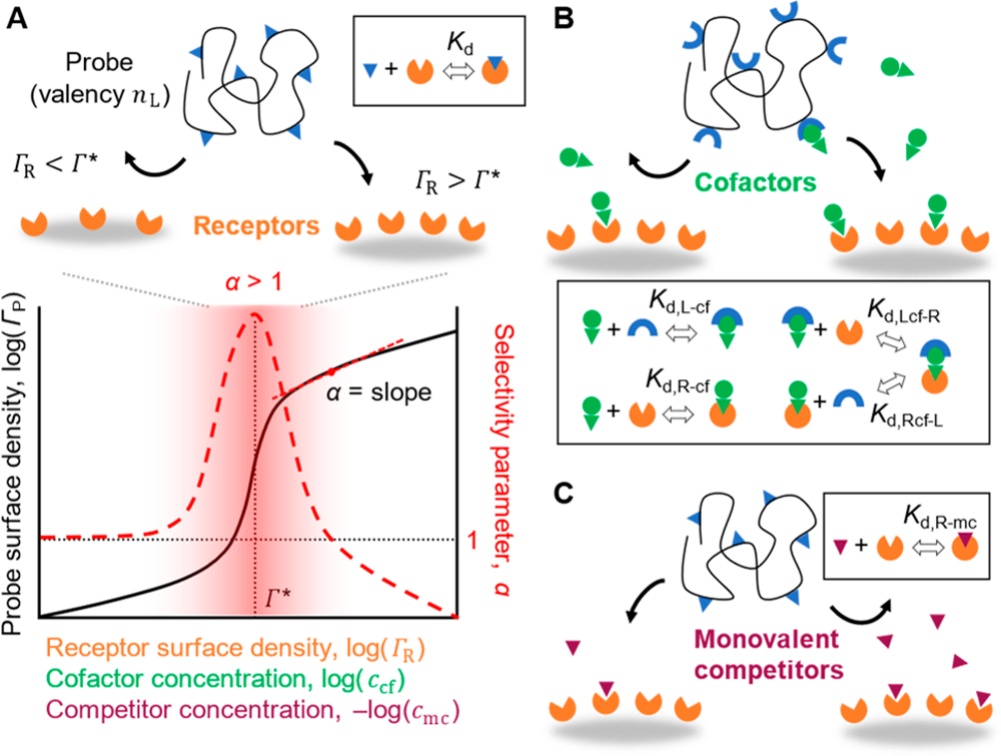
Multivalent interactions in the presence of competitors and cofactors. Superselectivity of multivalent probes to changes in receptor density (A, top) is modulated by the presence of cofactors (B) or competitors (C). Illustrative plot of probe surface density (solid black line) and corresponding selectivity parameters α (dashed red line) vs receptor density, and cofactor or competitor concentrations (A, bottom). Insets show the relevant reaction equilibria.

Previous studies of superselectivity in synthetic and living systems have clarified the roles of the affinity of individual ligand–receptor bonds, probe valency, receptor surface density, and in-plane mobility in multivalent binding.^2,14,17^ In addition to these factors, biological systems commonly involve interacting moieties that modulate multivalent interactions. For example, many specific interactions in biochemistry require a cofactor (e.g., a multivalent ion or a small molecule) to form a bond, and the strength of the interaction can be tuned by varying the concentration of cofactors.^7,18^ Likewise, competing interactions such as agonists vs antagonists are common in biology. The effect of cofactors (Figure 1B) or competitors (Figure 1C) on multivalent binding remains largely unexplored, hampering the wider application of superselectivity concepts. Cofactors and competitors modulate the effective number of available receptors, and we hypothesize that a superselective response toward changes in receptor density naturally extends to modulations in cofactor or competitor concentrations (Figure 1A, bottom).

Here, we demonstrate based on simple theoretical arguments that cofactors and monovalent competitors impact superselective binding by effectively rescaling the ligand–receptor affinity. We apply this insight to two important yet distinct examples of biomolecular interactions, namely, the Ca^2+^-dependent binding of the membrane repair protein annexin A5 (AnxA5) to anionic lipid membranes^7^ and the effect of competing oligosaccharides on the recognition of the extracellular matrix polysaccharide hyaluronan (HA) by cell surface receptors.^19^

The theory of multivalent binding^2,5,6^ predicts that the strength of the multivalent interaction, or avidity constant *K*_av_, depends supralinearly on the receptor density, Γ_R_, and the ligand–receptor dissociation constant, *K*_d_ (Figure 1A), as
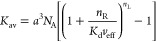
1where *a* and *n*_L_ are the size and valency of the multivalent probe, respectively, *n*_R_ = *a*^2^Γ_R_ the number of accessible receptors, *N*_A_ Avogadro’s number, and *v*_eff_ the effective free volume that each unbound ligand can explore (the ratio *n*_R_/*v*_eff_ is also called effective molarity^1,20^). When binding to a surface, this equation can be used as an input to the Langmuir isotherm, which predicts the surface density of adsorbed probes to be
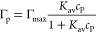
2with the maximum possible density Γ_max_ and the concentration of unbound probes *c*_P_. The binding is said to be superselective if the surface density increases faster than linearly with the receptor density, i.e., if the selectivity parameter
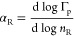
3is larger than unity (Figure 1A). Here we extend this theory to fully capture the effect of cofactors and competitors, including selectivity with regard to cofactor concentration *c*_cf_ (α_cf_ = d log Γ_p_/d log *c*_cf_) and competitor concentration *c*_mc_ (α_mc_ = −d log Γ_p_/d log *c*_mc_; the minus sign ensures that α_mc_ > 0, since binding generally decreases with *c*_mc_). The full theoretical derivation that considers the distribution of all possible binding states in equilibrium is provided in the Supporting Information, with only the main results being shown here.

## Cofactors

We consider monovalent cofactors at (unbound) concentration *c*_cf_ that bind to ligands and receptors with the dissociation constants *K*_d,L–cf_ and *K*_d,R–cf_, while the ligand–cofactor (or receptor–cofactor) complex binds to the receptors (or ligands) with constant *K*_d,Lcf–R_ (or *K*_d,Rcf–L_) (Figure 1B). The effect of cofactors can be fully captured by using a generalized ligand–receptor “affinity”, with an effective dissociation constant
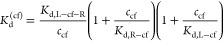
4where *K*_d,L–cf–R_ = *K*_d,L–cf_*K*_d,Lcf–R_ = *K*_d,R–cf_*K*_d,Rcf–L_ is the tripartite affinity constant.

**Figure 2:**
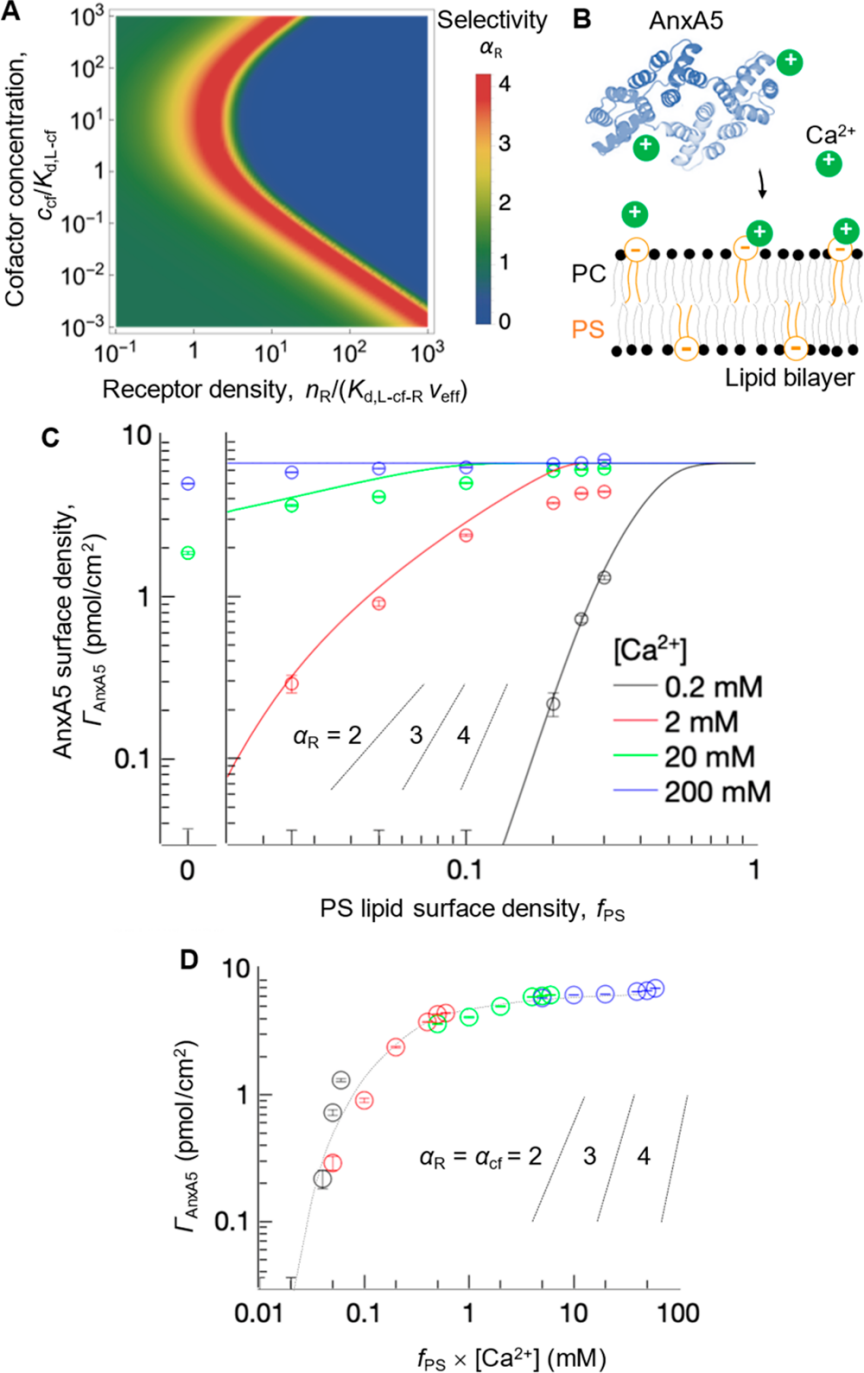
Effect of cofactors. (A) Example of the dependence of the selectivity parameter α_R_ on the receptor surface density and cofactor concentration (eqs 1–4; *n*_L_ = 8, *c*_P_*a*^3^*N*_A_ = 0.001, *K*_d,R–cf_ = 100*K*_d,L–cf_). (B) Schematic of AnxA5 (PDB code 1AVR(21)) binding to supported lipid bilayers presenting PS lipids in a background of PC lipids. (C) Experimental dependence of AnxA5 (nonoligomerizing mutant at *c*_P_ = 0.56 μM) binding on PS density at different Ca^2+^ concentrations (symbols; error bars represent experimental precision) is well reproduced by the theory (solid lines in matching colors) that explicitly models binding to the two types of lipids and membrane fluidity (see Supporting Information). (D) The sets of data at different Ca^2+^ concentration collapse onto a master curve when plotted as a function of *f*_PS_ × [Ca^2+^]. Slopes with α values are included in (C) and (D) for reference.

At low cofactor concentrations, *c*_cf_ < *K*_d,L–cf_ and *c*_cf_ < *K*_d,R–cf_, we can approximate *K*_d_^(cf)^ ≈ *K*_d,L–cf–R_/*c*_cf_, and thus changing the cofactor concentration has the same effect as changing the receptor density *n*_R_ (eq 1) and yields an equivalent superselective response (α_cf_ ≈ α_R_; Figure 1A, bottom). At intermediate concentrations, *K*_d,R–cf_ > *c*_cf_ > *K*_d,L–cf_ or *K*_d,L–cf_ > *c*_cf_ > *K*_d,R–cf_, either the ligands or receptors are saturated with cofactors, and changing the cofactor concentration has no effect: *K*_d_^(cf)^ ≈ max[*K*_d,Lcf–R_, *K*_d,Rcf–L_]. Lastly, at very high concentrations, *c*_cf_ > *K*_d,L–cf_ and *c*_cf_ > *K*_d,R–cf_, the oversaturation with cofactors weakens the effective binding: *K*_d_^(cf)^ ≈ *c*_cf_*K*_d,L–cf–R_/(*K*_d,R–cf_*K*_d,L–cf_), and thus changing *c*_cf_ has the same effect as changing the inverse receptor density *n*_R_^–1^ (Figure 2A). Often, however, only the low-concentration regime is biologically relevant. These features can be employed to control the range of superselective receptor recognition by tuning the cofactor concentration (Figure 2A). Thus, the influence of cofactors does not change the nature of multivalent binding; rather, it simply rescales the affinity constant according to eq 4.

A salient biological example of how cofactors influence multivalent interactions is AnxA5 binding to lipid membranes (Figure 2B). AnxA5 functions as a cell membrane scaffolding and repair protein.^22^ It preferentially binds anionic phospholipids and requires Ca^2+^ as a cofactor for membrane binding.^7^ In intact cells, anionic phospholipids reside in the inner (but not the outer) leaflet of the plasma membrane, whereas Ca^2+^ ions are virtually absent in the cytoplasm but present (in mM concentrations) outside the cell. AnxA5 thus binds to the cell membrane only upon membrane damage leading to an influx of Ca^2+^ ions into the cell and possibly also to interleaflet lipid content mixing near the damage site.

Experimental data reveal superselective binding of AnxA5 to lipid membranes presenting anionic phosphatidyl serine (PS) in a background of zwitterionic phosphatidyl choline (PC) lipids, and our theoretical model predicts well AnxA5 binding over 4 orders of magnitude of Ca^2+^ concentrations (Figure 2C). Moreover, within the range of the investigated calcium concentrations, the binding of Ca^2+^ to both AnxA5 and PS lipids appears to be weak: *c*_cf_/*K*_d,L–cf_ < 1 and *c*_cf_/*K*_d,R–cf_ < 1. Thus, eq 4 can be approximated as *K*_d_^(cf)^ = *K*_d,L–cf–R_/*c*_cf_, which implies that AnxA5 binding depends only on the product *n*_R_*c*_cf_ (eq 1), where *n*_R_ = *f*_PS_(*a*/*l*)^2^, with the protein cross-section *a*^2^ = 25 nm^2^, the lipid cross-section *l*^2^ = 0.7 nm^2^, and the PS lipid fraction *f*_PS_. Indeed, when the AnxA5 binding data are plotted as a function of *f*_PS_*c*_cf_, all experimental data collapse onto a single master curve (Figure 2D), thus validating our theory.

Our analysis identifies membrane recognition by AnxA5 as a striking example of superselective binding, demonstrating that binding is strongly superselective with respect to the cofactor Ca^2+^ as well as the receptor PS lipids, with maximal α values α_cf,max_ ≈ α_R,max_ ≈ 4 (Figure 2D). This enables the protein to effectively respond to slight changes in the concentration of either of these two factors, which is crucial for its function as a membrane repair protein. We note that effective membrane repair additionally requires AnxA5 to organize into trimers and two-dimensional crystals on the membrane.^22^ To probe superselective binding of the AnxA5 monomers, we have in Figure 2 probed an AnxA5 mutant that does not oligomerize yet retains the membrane binding properties of the wild-type protein. However, the superselective effects are retained, and even further accentuated, by the self-organization of the wild-type protein on the membrane (see Supporting Information).

## Competitors

Similar to the theoretical treatment of cofactors, monovalent competitors are assumed to bind to surface receptors with the affinity constant *K*_d,R–mc_. As shown in the full derivation of our analytical model, competitors at (unbound) concentration *c*_mc_ effectively rescale the ligand–receptor affinity *K*_d_ to
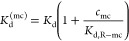
5

**Figure 3:**
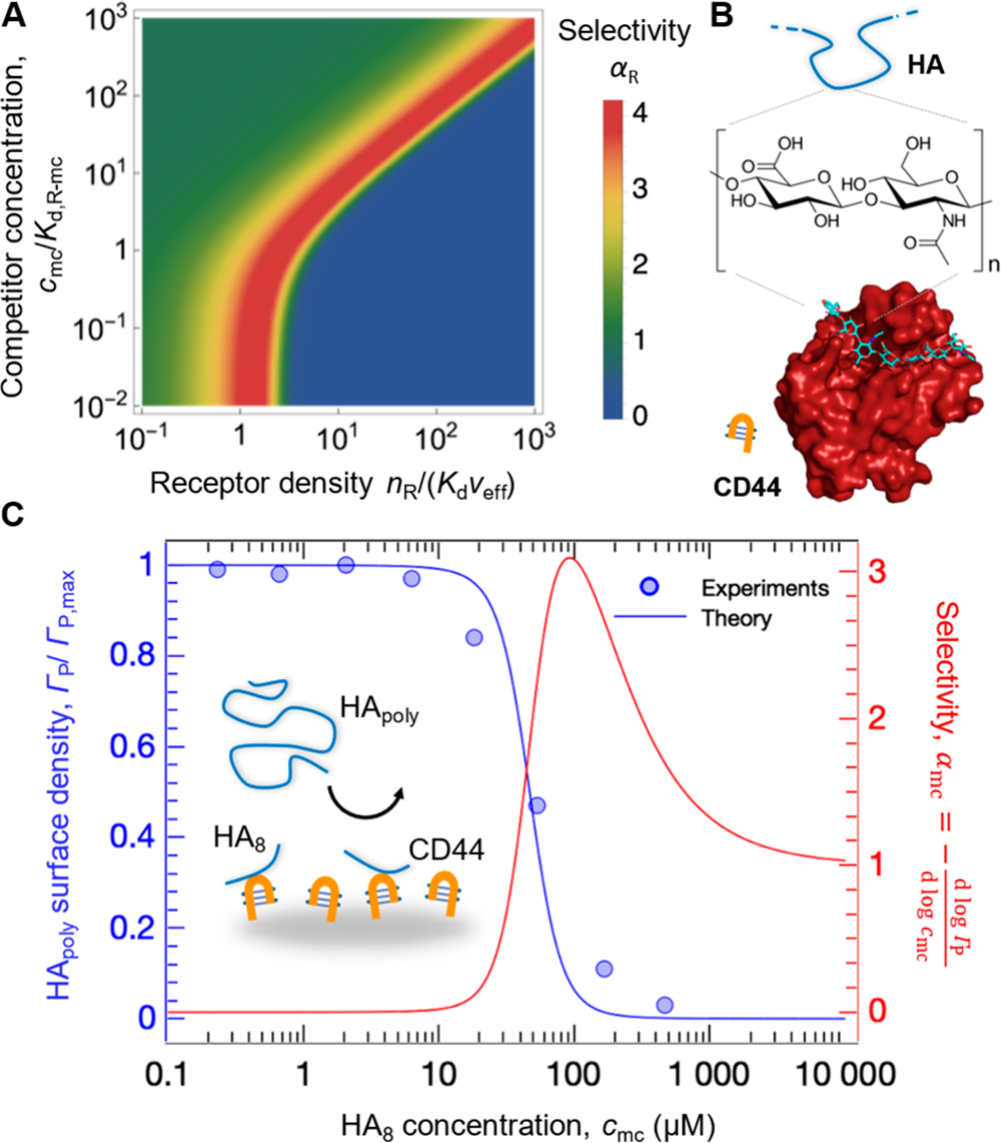
Effect of monovalent competitors. (A) Illustrative example of the dependence of the selectivity parameter α_R_ on the receptor surface density and competitor concentration (eqs 1–3, 5; *n*_L_ = 8, *c*_P_*a*^3^*N*_A_ = 0.001). (B) Schematic of HA binding to CD44 obtained from a crystal structure.^24^ (C) Competition of HA polysaccharides (HA_poly_) with octasaccharides (HA_8_) binding CD44 monovalently: experimental data from ref (19) (blue symbols), analytical fit (blue line), and the competitor selectivity α_mc_ (red line).

The impact of this rescaling on superselective binding is illustrated in Figure 3A and shows that increasing the competitor concentration pushes the range of superselective binding toward higher receptor densities. Equation 5 is well known for monovalent interactions;^29^ we here establish that it also applies to multivalent interactions and can be generalized to multiple competitor types (see Supporting Information).

We tested our simple model on data reported by Lesley et al.^19^ on the inhibition of HA polysaccharide binding to CD44 cell surface receptors by HA oligosaccharides (Figure 3B). That HA binding to cells depends sharply on receptor surface density is evident from previous work.^10,23^ Such superselective recognition is important for cell–extracellular matrix communication, and changes in HA presentation can dramatically affect recognition, e.g., inflammation entails degradation of large HA polysaccharides (MDa range) into small oligosaccharides. HA octasaccharides (HA_8_) just about fill the binding groove in a CD44 receptor^24^ and thus are effective monovalent competitors.

The simple analytical model (eqs 1 and 2) with the rescaled affinity *K*_d_^(mc)^ (eq 5) reproduces the experimental data well (Figure 3C), illustrating that it captures the salient features of the competition process. In the model, we fixed *n*_L_ = 500 distinct sites for binding to CD44 receptors (consistent with an HA molecular mass of ∼1 MDa and a decasaccharide “footprint” per receptor), a coil volume of *a*^3^ = 4*πR*_g_^3^/3 (with the radius of gyration, *R*_g_ ≈ 90 nm,^25^ and a concentration of *c*_P_ ≈ 0.5 nM), and *K*_d,R–mc_ ≈ 50 μM (within the broad range of reported values^19,24,26^). As the only fitting parameter, we determined *n*_R_/(*K*_d_*v*_eff_) ≈ 0.03, a value that is consistent with typical CD44 cell surface densities (see Supporting Information); that is, the simple model makes reasonable quantitative predictions.

Importantly, we demonstrate that the binding response can be superselective with respect to the competitor concentration *c*_mc_ (α_mc_ > 1; Figure 3C). The fact that the experimental dependence is less sharp then predicted theoretically is attributed to the relatively large polydispersity of HA polymers (ranging from 0.5 to 3 MDa) used in the experiments,^19^ which is not considered by the analytical model.

The above reanalysis of data from the literature demonstrates the tangible benefits of superselectivity concepts. It is well known that small vs large HA can exert opposing functional effects,^27^ but the underpinning mechanisms have long remained elusive. With the theoretical tool presented here, we can rationalize how HA molecules of different sizes bind and compete with each other for receptors. Moreover, we can predict how changes in the presentation of HA (e.g., the effective mean size and size dispersity, which may be modulated by degradation or by cross-linking with soluble HA binding proteins) and its receptors (e.g., their affinity, surface density, and clustering) modulate HA binding and downstream physiological processes.

In conclusion, we have developed a new mechanistic understanding of multivalent recognition with cofactors and competitors. Rather than modifying the multivalent probe itself, the addition of monovalent binders as competitors or cofactors is a simple, and thus attractive, avenue to modulate superselective binding. This effect can be exploited, for example, to tune the threshold receptor density Γ* of a given probe (Figure 3A), to target surfaces with low receptor density,^28^ and for superselective discrimination of cofactor concentrations (Figure 2D). Our theory thus helps design superselective probes for targeting and analytical purposes controlled by cofactors and competitors. While the simple multivalent model (eqs 1 and 2) assumes each ligand can bind to many receptors, the scaling expressions (eqs 4 and 5) are general: they expand on similar and well-known expressions for monovalent interactions^29^ and also apply to systems with few receptors and many ligands (see Supporting Information).

## Supporting Information Available

Theoretical derivations, details of the AnxA5-to-membrane binding experiments and binding model, and details of the HA-to-CD44 binding analysis (PDF)
