# Supplementary material for: Controlling Superselectivity of Multivalent Interactions with Cofactors and Competitors
Source: J Am Chem Soc. 2022 Sep 14;144(38):17346–50. doi: 10.1021/jacs.2c06942 (PMC9523699; doi:10.1021/jacs.2c06942)
Supplement: Supplementary file 1 — ja2c06942_si_001.pdf [file ja2c06942_si_001.pdf]

Controlling superselectivity of multivalent interactions with cofactors and competitors

Tine Curk,[¶,] Galina V. Dubacheva,[¶] Alain R. Brisson, and Ralf P. Richter**

Supporting Information

Contents

1 Experimental Methods 2
1.1 Expression and purification of non-oligomerising annexin A5 mutant protein 2
1.2 Quantitation of annexin A5 binding to supported lipid bilayers 2
2 Supporting Theoretical Methods 5
2.1 Analytical model for the binding of multivalent probes requiring cofactors 5
2.2 Analytical model for binding of Annexin A5 to lipid membranes 6
2.3 Analytical model for binding of multivalent probes in the presence of monovalent competitors 9
2.4 HA binding to CD44-presenting cells – Estimating CD44 surface densities from the analysis of HA polysaccharide binding in competition with HA_8 11
3 Supporting References 11

1 EXPERIMENTAL METHODS

1.1 Expression and purification of non-oligomerising annexin A5 mutant protein

To produce the (R16E, R23E, K27E, K56E, K191E) annexin A5 mutant (AnxA5 mut) protein, the AGG, CGG, AAA, AAG and AAG codons were replaced by GAG for R16E, R23E, K27E, K56E and K191E, respectively. Mutations were verified by double-strand DNA sequence analysis. AnxA5 mut was expressed in *E. coli* BL21(DE3) cells and purified as previously described.[1] The protein stock solutions had a concentration of ~1 mg/mL and were stored at 4 °C.

In contrast to wild type AnxA5 (AnxA5 WT), AnxA5 mut does not form trimers nor two-dimensional arrays of trimers upon membrane binding. However, the tertiary structure and membrane-binding interface of the protein are preserved ensuring that AnxA5 mut binds PS-containing membranes similarly to AnxA5 WT.[1-2]

1.2 Quantitation of annexin A5 binding to supported lipid bilayers

In an earlier study,[3] we had systematically analyzed the binding of AnxA5 WT to supported lipid bilayers (SLB) made of PC and PS lipids by quartz crystal microbalance (QCM-D). We here considered the QCM-D frequency shifts reported in Ref. 3 for equilibrium binding of AnxA5 WT at a concentration of 0.56 µM to silica-supported lipid bilayers as well as mica-supported lipid bilayers, as a function of the molar fraction of PS in the surface-distal SLB leaflet (i.e., the leaflet accessible for AnxA5 binding) and the Ca^{2+} concentration in the solution phase. These data are reproduced in Figure S1B-C for the reader's convenience.

Our earlier study[3] also demonstrated that AnxA5 WT forms mostly trimers upon binding to silica-SLBs. The trimers additionally self-organize into two-dimensional crystals on mica-SLBs; low-density crystals (p6 symmetry) start to form at low protein surface coverages (<10%) and a phase transition into a denser crystal (p3 symmetry) occurs once the surface coverage exceeds the capacity of the p6 crystal form.

The binding of AnxA5 mut to silica-SLBs made of PC and PS lipids was here assayed analogously, by QCM-D, following the procedures described in Ref. 3 (Figure S1A). Taken together, the interaction scenarios covered enable us to probe the multivalent and Ca^{2+}-mediated binding of AnxA5 monomers to anionic lipid membranes (AnxA5 mut on silica-SLBs) as well as the effects of protein oligomerization (AnxA5 WT on silica-SLBs) and protein 2D crystallization (AnxA5 WT on mica-SLBs).

QCM-D frequency shifts (Δf) were converted to protein surface densities (Γ) using previously established standard curves for AnxA5 WT and AnxA5 mut on silica-SLBs (Figure S1D, *colored symbols* and *dashed lines*).[4] This approach implicitly corrects for the contribution of hydrodynamically coupled solvent to the QCM-D frequency shifts, which depends non-linearly on coverage for protein monomers or oligomers that are evenly distributed across the surface.[5] For extended crystals, on the other hand, the relative contribution of coupled solvent to the QCM-D frequency shifts is expected to be independent of coverage.[4] This approximation was used to establish a standard curve to convert Δf into Γ for AnxA5 WT on mica-SLBs (Figure S1D, *black dashed line*).

The equilibrium binding curves of AnxA5 mut as a function of the molar fraction of PS (f_{PS}) at constant Ca^{2+} concentration (c_{cf}) are shown in Figure 2C. Figure S2 shows the same data, but

as a function of c_{cf} at constant f_{PS}. Binding is superselective with respect to f_{PS} ($\alpha_{\mathrm{R}} > 1$) as well as c_{cf} ($\alpha_{\mathrm{cf}} > 1$).

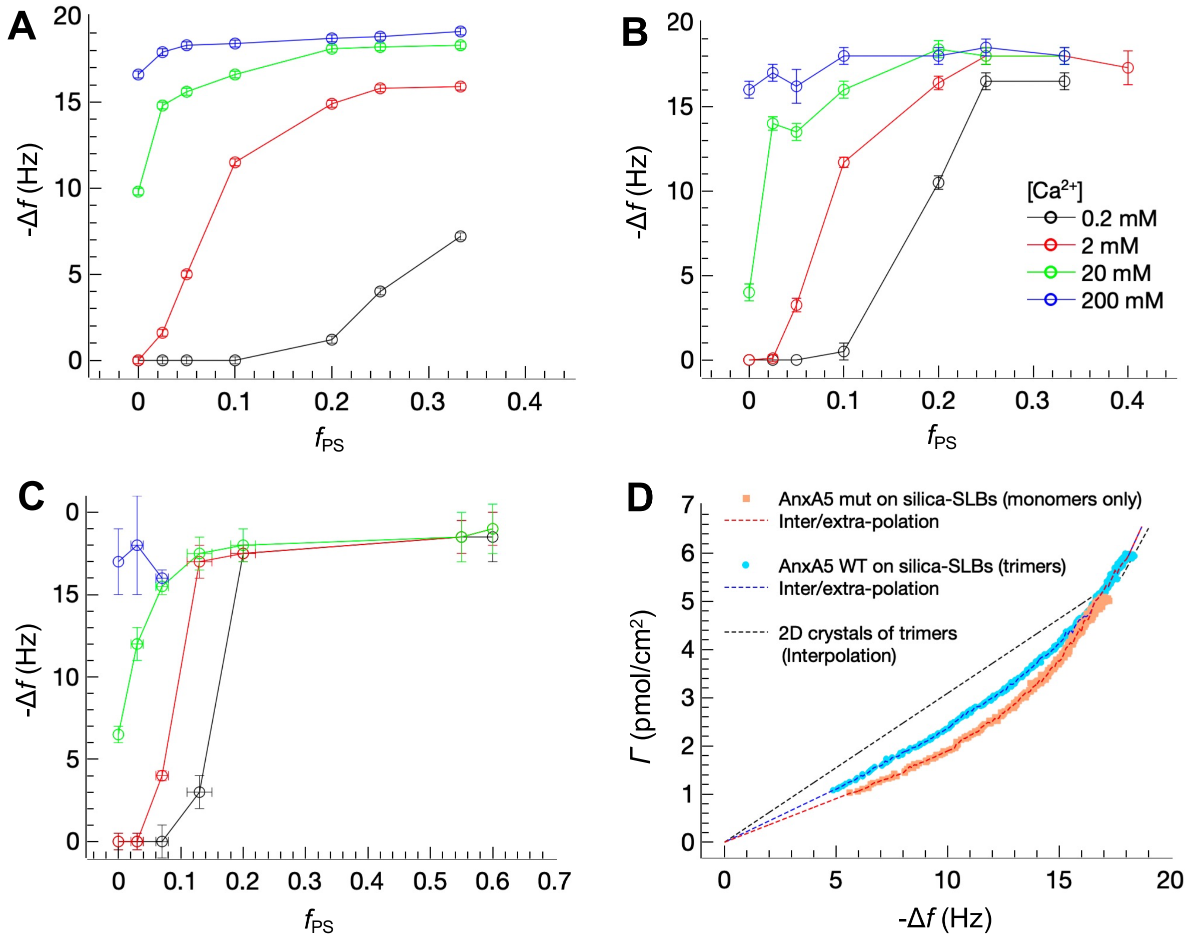

Figure S1. Quantitation of AnxA5 surface densities from QCM-D frequency shifts. **(A)** QCM-D frequency shifts at equilibrium for the binding of 0.56 μM AnxA5 mut (with impaired oligomerization) to silica-supported lipid bilayers as a function of the molar fraction of PS (f_{PS}) in the surface-distal (and thus AnxA5 accessible) SLB leaflet, for several Ca^{2+} concentrations (as indicated, with *color code*). **(B-C)** Equivalent data for AnxA5 WT on silica-supported lipid bilayers (B) and mica-supported lipid bilayers (C), taken from Figures 3 and 6E-H in Ref. 3, respectively. AnxA5 WT forms trimers on silica-SLBs and additionally crystallizes in two-dimensions on mica-SLBs.[3] **(D)** Experimental data for the binding of AnxA5 mut (*orange dots*) and AnxA5 WT (*blue dots*) to silica-supported lipid bilayers, taken from Ref. 4; these data were obtained with a setup that combined QCM-D and spectroscopic ellipsometry *in situ* to simultaneously assess frequency shifts (Δf) and molar surface densities (Γ), respectively, during AnxA5 binding to silica-SLBs containing 25 mol-% PS. Dashed lines in darker matching colors are interpolations up to $-\Delta f$ = 18.0 Hz (for AnxA5 WT) and 16.4 Hz (for AnxA5 mut), and linear extrapolations beyond (up to 18.7 Hz). The black dashed line for two-dimensional crystals is based on Ref. 6, assuming p6 crystals up to 17.5 Hz and then gradual conversion into p3 crystals until a full p3 crystalline layer is formed at 19 Hz, and a linear relationship between $-\Delta f$ and Γ for each of the two crystallization phases (see page S2 for the rationale of this approach). The interpolated/extrapolated curves were used to convert equilibrium AnxA5 frequency shifts (Δf) to molar surface densities (Γ), as shown in Figure 2 and Figures S2 and S3.

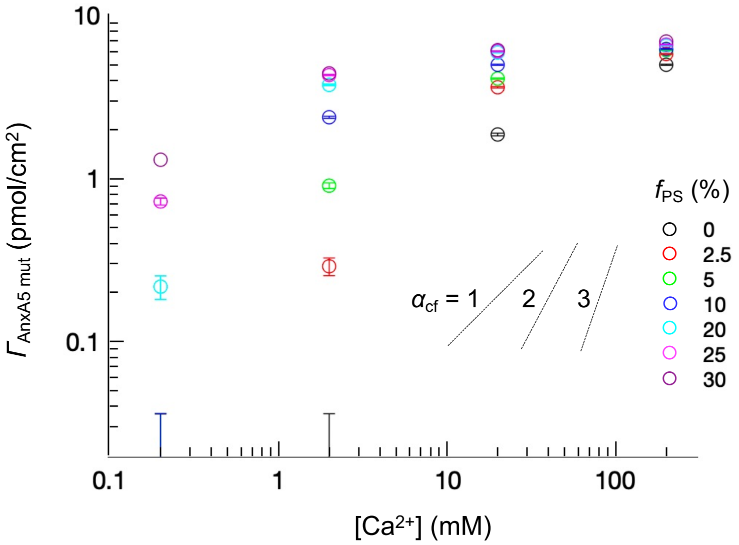

Figure S2. Binding of AnxA5 mut – dependence on Ca^{2+} concentration. Experimental data of the dependence of AnxA5 (non-oligomerizing mutant) equilibrium binding on c_{cf} at different f_{PS} (*symbols*; *error bars* represent experimental precision). Slopes of 1, 2 and 3 are shown for reference, and demonstrate that the selectivity parameter α_{cf} reaches values of at least 2. Our theoretical model suggests that higher values of $\alpha_{\mathrm{cf}} \approx 4$ are reached (see main text for details), but this is not fully resolved in the experimental data because a relatively small set of Ca^{2+} concentrations were tested. Conditions that showed no measurable interactions are indicated by an error bar indicating the detection limit; note that the data at 0.2 mM Ca^{2+} superpose for f_{PS} = 0, 2.5, 5 and 10% (below the detection limit). The same data are plotted in Figure 2C as a function of f_{PS}, at different c_{cf}, evidencing $\alpha_{\mathrm{R}} \approx 4$.

2 SUPPORTING THEORETICAL METHODS

We generally use the terms 'ligand' and 'receptor' to denote binding partners on the multivalent probe and on the surface, respectively, irrespective of whether these are ligands or receptors from a biological point of view.

2.1 Analytical model for the binding of multivalent probes requiring cofactors

To derive the effect of cofactors we calculate the partition function Q, which is a sum over all possible binding states. The ratio between the partition function over *bound* states Q_b (probe attached to a surface lattice site) and *unbound* states Q_u (probe free in solution), determines the interaction free energy F via $Q_\mathrm{b}/Q_\mathrm{u} = e^{-F/k_\mathrm{B}T}$ and thus the avidity of probe–membrane binding.[7-8] Cofactors, at concentration c_cf, are here considered necessary to form a ligand–cofactor–receptor bond. The Gibbs free energy for the ligand–cofactor–receptor complex formation is $\Delta G_\mathrm{L-cf-R}$ and the associated affinity constant for the formation of a tripartite complex from a ligand, a cofactor and a receptor is $K_\mathrm{d,L-cf-R} = \rho_0^2 e^{\Delta G_\mathrm{L-cf-R}/k_\mathrm{B}T}$. We consider that the cofactor can bind to a receptor with affinity $K_\mathrm{d,R-cf}$ and to a ligand with affinity $K_\mathrm{d,L-cf}$. The receptor–cofactor complex can then bind to a ligand with affinity $K_\mathrm{d,Rcf-L} = K_\mathrm{d,L-cf-R}/K_\mathrm{d,R-cf}$. Conversely, the ligand–cofactor complex can bind to a receptor with affinity $K_\mathrm{d,Lcf-R} = K_\mathrm{d,L-cf-R}/K_\mathrm{d,L-cf}$. To calculate the partition sum of bound states, we also consider that every ligand on the multivalent probe can be either attached to a receptor (with Gibbs free energy $\Delta G_\mathrm{L-cf-R}$) or free to explore a molar volume v_eff.[7-8] In addition, we require that at least one ligand–receptor bond is present for the probe to be considered bound. The partition sum thus reads,

$$Q_\mathrm{b} = v_\mathrm{b} \sum_{i=1}^{\min[n_\mathrm{L},n_\mathrm{R}]} \Omega(i) \left(\frac{c_\mathrm{cf} e^{-\Delta G_\mathrm{L-cf-R}/k_\mathrm{B}T}}{\rho_0}\right)^i (v_\mathrm{eff}\rho_0)^{n_\mathrm{L}-i} \left(1 + \frac{c_\mathrm{cf}}{K_\mathrm{d,R-cf}}\right)^{n_\mathrm{R}-i} \left(1 + \frac{c_\mathrm{cf}}{K_\mathrm{d,L-cf}}\right)^{n_\mathrm{L}-i}, \quad \text{[S1A]}$$

where $\Omega(i)$ is the number of distinct states to form i bonds between ligands and receptors, and each formed ligand–receptor bond requires recruitment of a cofactor from solution (hence the c_cf/ρ_0 factor, where c_cf is the concentration of unbound cofactors). In addition, every receptor that is not occupied by a ligand (number $n_\mathrm{R} - i$) can be independently either free (weight 1) or attached to a cofactor (weight $c_\mathrm{cf}/K_\mathrm{d,R-cf}$), hence the additional term, $\left(1 + c_\mathrm{cf}/K_\mathrm{d,R-cf}\right)^{n_\mathrm{R}-i}$. Likewise, every ligand that is not bound to a receptor can be either free (weight 1) or attached to a cofactor (weight $c_\mathrm{cf}/K_\mathrm{d,L-cf}$), yielding an additional term, $\left(1 + c_\mathrm{cf}/K_\mathrm{d,L-cf}\right)^{n_\mathrm{L}-i}$.

The pre-factor v_b considers that the center-of-mass of the multivalent probe is allowed to move within a lattice volume v_b when bound to a surface. This volume is determined by the lattice size a used with the Langmuir adsorption model (Eq. [2]), $v_\mathrm{b} \sim a^3$, where a is the typical size of the multivalent probe. Moreover, any nonspecific interactions between the probe and the surface should be included in this pre-factor, i.e., any interaction that does not depend on the number of formed ligand-receptor bonds, such as hydrophobic attraction, or steric repulsion if the probe is a polymer.[9] If the strength of this interaction is U_ns, the pre-factor becomes $v_\mathrm{b} \sim a^3 \exp(-U_\mathrm{ns}/k_\mathrm{B}T)$. Lastly, if the probe can bind to the surface only with a specific orientation, such as a multivalent protein binding to a membrane, the loss of orientational degrees of freedom must be captured by the pre-factor.

The unbound partition function is obtained by summing over all states where ligand–receptor binding does not occur as the probe is in solution. Each ligand is unbound, can explore a volume v_{eff} and can independently bind to a cofactor. Likewise, all n_{R} receptors can independently bind to a cofactor. The partition function reads

$$Q_{\mathrm{u}} = (\rho_0 N_{\mathrm{A}})^{-1} (v_{\mathrm{eff}}\rho_0)^{n_{\mathrm{L}}} \left(1 + \frac{c_{\mathrm{cf}}}{K_{\mathrm{d,R-cf}}}\right)^{n_{\mathrm{R}}} \left(1 + \frac{c_{\mathrm{cf}}}{K_{\mathrm{d,L-cf}}}\right)^{n_{\mathrm{L}}}, \qquad \text{[S1B]}$$

where the pre-factor $(\rho_0 N_{\mathrm{A}})^{-1}$ determines the reference chemical potential of free moieties, with the standard value $\rho_0 = 1$ M and Avogadro's number N_{A}.

The ratio of these two partition functions determines the free energy of the multivalent interaction,

$$e^{-F/k_{\mathrm{B}}T} = \frac{Q_{\mathrm{b}}}{Q_{\mathrm{u}}} = v_{\mathrm{b}}\rho_0 N_{\mathrm{A}} \sum_{i=1}^{\min[n_{\mathrm{L}}, n_R]} \Omega(i) \left[\frac{c_{\mathrm{cf}}\, e^{-\Delta G_{\mathrm{L-cf-R}}/k_{\mathrm{B}}T}}{v_{\mathrm{eff}}\rho_0{}^2\left(1+\frac{c_{\mathrm{cf}}}{K_{\mathrm{d,R-cf}}}\right)\left(1+\frac{c_{\mathrm{cf}}}{K_{\mathrm{d,L-cf}}}\right)}\right]^i. \qquad \text{[S2]}$$

This expression reduces to the standard case without cofactors ($c_{\mathrm{cf}} = 0$) by defining a rescaled affinity constant

$$K_{\mathrm{d}}^{(\mathrm{cf})} = \frac{K_{\mathrm{d,L-cf-R}}}{c_{\mathrm{cf}}}\left(1 + \frac{c_{\mathrm{cf}}}{K_{\mathrm{d,R-cf}}}\right)\left(1 + \frac{c_{\mathrm{cf}}}{K_{\mathrm{d,L-cf}}}\right). \qquad \text{[S3]}$$

Thus, the effect of the cofactors does not change the nature of multivalent binding, it only rescales the affinity constant according to Eq. [S3]. Crucially, this rescaling is general for any form of $\Omega(i)$ and thus for any type of multivalent interaction if the binding of cofactors can be considered independent, i.e., there are no allosteric effects, and thus the K_{d} values can be considered constants.

We note that cofactors could themselves be multivalent in the sense that a cofactor can bind more than one receptor, e.g., one ion binding two lipids in the case of membrane binding proteins. If the number of receptors is large, such that receptors are not depleted, Eq. [4] in the main text still holds but the receptor-cofactor dissociation constant $K_{\mathrm{d,R-cf}}$ is not a simple constant and is instead determined by the avidity of a multivalent co-factor binding to a surface. Equivalent arguments can be made for cofactors that bind to more than one ligand. However, for the sake of simplicity we do not consider these more complex scenarios quantitatively here.

2.2 Analytical model for binding of Annexin A5 to lipid membranes

AnxA5 binds strongly to membranes containing negatively charged PS lipids, but it also binds (albeit more weakly) to membranes made purely of zwitterionic PC lipids.[3, 10] In either case, binding requires a Ca^{2+} ion as cofactor,[3, 10] and based on structural analyses each AnxA5 monomer is believed to have at least 8 lipid binding sites. Moreover, the lipids are mobile in the membrane plane. This scenario requires a more complicated model where we consider the molar fraction of PS lipids f_{PS} in the membrane (the fraction of PC lipids is $f_{\mathrm{PC}} = 1 - f_{\mathrm{PS}}$). At the calcium concentrations considered, the AnxA5 experimental system falls in the regime where the binding of the cofactor alone to the surface lipids is very weak ($K_{\mathrm{d,R-cf}} \gg c_{\mathrm{cf}}$). Likewise, since experimental data show that the binding of AnxA5 is strongly c_{cf} dependent even at the highest c_{cf} concentrations, we conclude that the binding of the cofactors to the

protein is also weak ($K_{\mathrm{d,L-cf}} > c_{\mathrm{cf}}$). Thus, we simplify Eq. [S3] by only keeping the leading term,

$$K_{\mathrm{d}}^{(\mathrm{cf})} = K_{\mathrm{d,L-cf-R}}/c_{\mathrm{cf}}. \qquad \text{[S4]}$$

To obtain the free energy of binding a single AnxA5 molecule to a membrane we consider binding to the PS lipids with Gibbs free energy $\Delta G_{\mathrm{L-cf-R,PS}}$ and to the PC lipids with Gibbs free energy $\Delta G_{\mathrm{L-cf-R,PC}}$. Note that these free energies pertain to protein–cofactor–lipid complex formation via Ca^{2+} as the cofactor. We consider that each Annexin A5 binding site can be free or attached either to a PS or PC lipid via a cofactor at concentration c_{cf}. For a fluid membrane and multiple 'receptor' types,[8] and using the approximation in Eq. [S4], the free energy of AnxA5 binding to a membrane is given by

$$e^{-F/k_{\mathrm{B}}T} = v_{\mathrm{b}}\rho_0 N_{\mathrm{A}}\left\{\left[1+\frac{c_{\mathrm{cf}}}{v_{\mathrm{eff}}{\rho_0}^2}\left(n_{\mathrm{R,PS}}e^{-\Delta G_{\mathrm{L-cf-R,PS}}/k_{\mathrm{B}}T}+n_{\mathrm{R,PC}}e^{-\Delta G_{\mathrm{L-cf-R,PC}}/k_{\mathrm{B}}T}\right)\right]^{n_{\mathrm{L}}}-1\right\}, \qquad \text{[S5]}$$

where $n_{\mathrm{R,PS}}$ and $n_{\mathrm{R,PC}}$ are the numbers of free PS and PC lipids, respectively, covered by each AnxA5. Since the membrane under consideration is composed only of these two lipid types, we can express $n_{\mathrm{R,PS}} = \hat{f}_{\mathrm{PS}}\, A_{\mathrm{P}}/A_{\mathrm{L}}$ and $n_{\mathrm{R,PC}} = \left(1-\hat{f}_{\mathrm{PS}}\right)A_{\mathrm{P}}/A_{\mathrm{L}}$ in terms of the fraction of free PS lipids $\hat{f}_{\mathrm{PS}}$ where A_{L} is the membrane surface area occupied by a single lipid and A_{P} the surface area occupied by an AnxA5 protein. Note that, due to depletion of free PS lipids upon binding to AnxA5, $\hat{f}_{\mathrm{PS}} \leq f_{\mathrm{PS}}$, where f_{PS} is the overall fraction of PS lipids and is a constant. We combine all unknown parameters and define rescaled binding free energies,

$$e^{\hat{G}_{\mathrm{PS}}/k_{\mathrm{B}}T} = v_{\mathrm{eff}}\,\frac{A_{\mathrm{L}}}{A_{\mathrm{P}}}\rho_0 e^{\Delta G_{\mathrm{L-cf-R,PS}}/k_{\mathrm{B}}T}, \text{ and} \qquad \text{[S6A]}$$

$$e^{\hat{G}_{\mathrm{PC}}/k_{\mathrm{B}}T} = v_{\mathrm{eff}}\,\frac{A_{\mathrm{L}}}{A_{\mathrm{P}}}\rho_0 e^{\Delta G_{\mathrm{L-cf-R,PC}}/k_{\mathrm{B}}T}. \qquad \text{[S6B]}$$

Inserting Eqs. [S6A-B] into Eq. [S5], we obtain an expression that only contains two fitting parameters ($\hat{G}_{\mathrm{PS}}$ and $\hat{G}_{\mathrm{PC}}$),

$$e^{-F/k_{\mathrm{B}}T} = v_{\mathrm{b}}\rho_0 N_{\mathrm{A}}\left\{\left[1+\frac{c_{\mathrm{cf}}}{\rho_0}\left(\hat{f}_{\mathrm{PS}}e^{-\hat{G}_{\mathrm{PS}}/k_{\mathrm{B}}T}+(1-\hat{f}_{\mathrm{PS}})e^{-\hat{G}_{\mathrm{PC}}/k_{\mathrm{B}}T}\right)\right]^{n_{\mathrm{L}}}-1\right\}. \qquad \text{[S7]}$$

$\hat{f}_{\mathrm{PS}}$ is determined by a self-consistent solution that preserves the total number of lipids in the membrane.[11] Moreover, we use $A_{\mathrm{L}} = 0.7$ nm^2 for the typical cross-section of the lipids in the membrane plane, and $A_{\mathrm{P}} = a^2$ with $a = 5$ nm taken from the size of AnxA5.[3, 6] We note that the pre-factor v_{b} includes contributions from non-specific binding (e.g., hydrophobic) and from the loss of orientational freedom when binding to the membrane. These two effects are expected to partially cancel out and thus we use a simple estimate, $v_{\mathrm{b}} \approx a^3$. Changing this prefactor by a factor of 10 does not noticeably affect the goodness of the fit, it merely changes the bond energies $\hat{G}_{\mathrm{PS}}$ and $\hat{G}_{\mathrm{PC}}$ (the binding energies scale with $\ln(v_{\mathrm{b}})$ for weak binding according to first-order Taylor expansion of Eq. [S7], i.e., $(1+x)^n \approx 1+nx$ for $x \ll 1$).

We compared the theoretical predictions with experimental data for the AnxA5 mutant with impaired oligomerization capacity. To determine the surface coverage and compare with experimental data, we use the Langmuir isotherm (Eq. [2]) at AnxA5 concentration $c_{\mathrm{P}} = 0.56$ µM and assume maximum molar coverage is determined by a fully packed surface, $\Gamma_{\max} = (A_{\mathrm{P}}N_{\mathrm{A}})^{-1}$. We further assumed, in a first instance, the number of lipid binding sites per protein to be $n_{\mathrm{L}} = 8$.[10] The two unknown parameters are globally fitted using a least-squares fit in the

logarithmic measure, to $\hat{G}_{\mathrm{PS}} = -11.2\,k_{\mathrm{B}}T$ and $\hat{G}_{\mathrm{PC}} = -5.3\,k_{\mathrm{B}}T$. Overall, the theory reproduced very well the superselective binding of AnxA5 mut and the effect of cofactors over a wide range of receptor and cofactor concentrations (Fig. 2). Small quantitative discrepancies remain though which we attribute to residual interactions between membrane-bound AnxA5 molecules, and not all 8 lipid binding sites per AnxA5 molecule being perfectly equivalent (which is not considered by the theory).[10]

Moreover, our estimation of $\hat{G}_{\mathrm{PS}}$ is broadly consistent with published data on total AnxA5–membrane binding of $F = 89\ k_{\mathrm{B}}T$ (equivalent to 53 kcal/mol) at $f_{\mathrm{PS}} = 0.25$ and standard conditions (i.e., $c_{\mathrm{cf}} = \rho_0 = 1$ M).[10] At these conditions we find $F = 79\ k_{\mathrm{B}}T$. Another report[12] found a value $F = 38\ k_{\mathrm{B}}T$ at $f_{\mathrm{PS}} = 0.2$ and $c_{\mathrm{cf}} = 2$ mM (i.e., close to physiologically relevant calcium concentrations), and our model predicts $F = 27\ k_{\mathrm{B}}T$. Our predicted values, obtained for the AnxA5 mutant, are consistently ~10 $k_{\mathrm{B}}T$ lower than previously published values on wild type AnxA5. The enhanced affinity of the wild type protein may be attributed to AnxA5 oligomerization (and crystallization) on the membrane, which is suppressed in the mutant.

We also attempted to fit the data assuming $n_{\mathrm{L}} = 4$, corresponding to one lipid binding site per each of the 4 homologous domains in AnxA5.[13-14] However, this resulted in a poorer fit with a root-mean-square error increased by ~30%. In contrast, $n_{\mathrm{L}} > 8$ improved the fit compared to $n_{\mathrm{L}} = 8$, confirming that AnxA5 has indeed a remarkably high valency for lipid binding,[10, 15] although with our simple model we are unable to discriminate the exact number of lipid binding sites per protein. We note in passing that the scaling of AnxA5 binding with $f_{\mathrm{PS}}c_{\mathrm{cf}}$ (Figure 2D) indicates that each AnxA5-lipid interaction requires one Ca^{2+} ion as cofactor. For two Ca^{2+} ions per lipid (as proposed in some previous works[14]), AnxA5 binding should instead scale with $f_{\mathrm{PS}}c_{\mathrm{cf}}^2$, which is incompatible with our data.

We also considered the cases of the wild type AnxA5 protein binding to silica-supported lipid bilayers (where it forms trimers) and to mica-supported lipid bilayers (where it forms two-dimensional crystals of trimers). Figure S3 reviews the experimental data for the wild-type AnxA5 surface densities as a function of f_{PS} for a range of Ca^{2+} cofactor concentrations (taken from Ref. 3, and adapted as described in Figure S1). When compared to the AnxA5 mutant data (Figures 2 and S2), we find the superselective recognition with respect to receptors and cofactors is preserved, and even further enhanced. Attempts to reproduce the data with the above-described model did not result in a satisfactory fit (not shown). This can be qualitatively appreciated from Figure S3B, where the data for the lowest Ca^{2+} cofactor concentration (0.2 mM) do not fall on a master curve with the data for the three other cofactor concentrations. We tested a more complex model that explicitly considered the AnxA5 trimerization step in the statistical mechanics analysis: this improved the fit somewhat but remained relatively poor (not shown). A possible explanation is that AnxA5 oligomerization is itself reliant on Ca^{2+} ions as a cofactor.

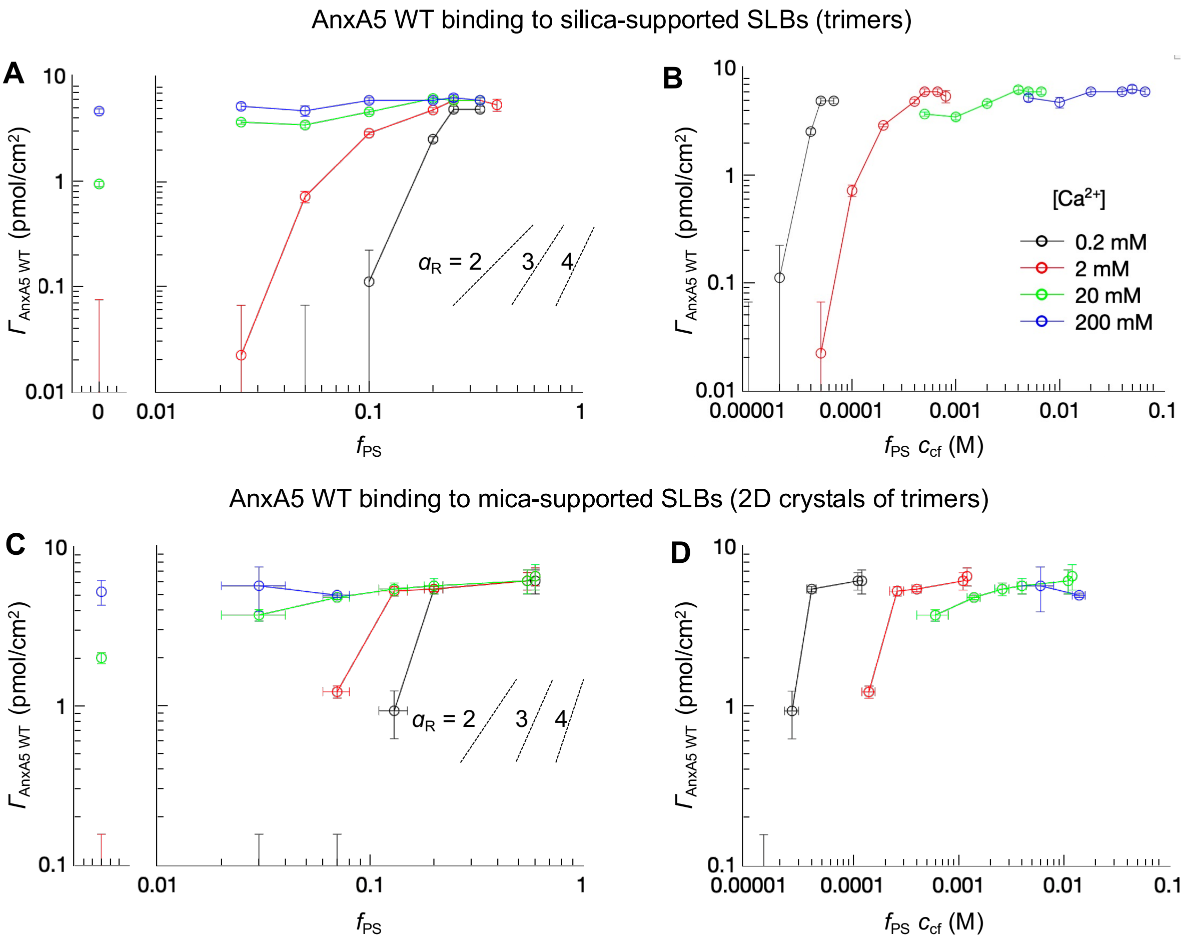

Figure S3. Superselective recognition of anionic lipid membranes and Ca^{2+} ions by Annexin A5 wild type protein. Whilst the tertiary structure and membrane-binding interface are essentially preserved in the AnxA5 mut, AnxA5 WT additionally self-organizes upon membrane binding. On silica-supported lipid bilayers, the self-organization is largely limited to the formation of trimers except at very high coverages when two-dimensional crystallization into a densely packed p3 lattice might occur.[3] On mica-supported lipid bilayers, the trimers additionally organize into two-dimensional crystals. We here show that trimer formation and two-dimensional crystallization preserve, and even slightly enhance, the quality of superselective binding. **(A)** AnxA5 WT surface density on silica-supported lipid bilayers presenting phosphatidyl serine (PS) in a background of phosphatidyl choline (PC) lipids, at different concentrations c_{cf} of the Ca^{2+} cofactor (*symbols with error bars*). **(C)** Equivalent data for AnxA5 WT on mica-supported lipid bilayers. Data were obtained from Figure S1, and the AnxA5 concentration was 0.56 µM in all assays. Slopes of 2, 3 and 4 are shown for reference, and demonstrate that superselectivity parameters of $\alpha_{\mathrm{R}} \approx 4$ are reached for the lower Ca^{2+} concentrations. **(B, D)** In contrast to AnxA5 mut (Figure 2D), the four sets of data at different cofactor (Ca^{2+}) concentration do not collapse onto a master curve when plotted as a function of $f_{\mathrm{PS}}c_{\mathrm{cf}}$, where f_{PS} is the molar fraction of negatively charged PS lipids in the membrane ($f_{\mathrm{PS}} \propto n_{\mathrm{R}}$). A possible explanation is that AnxA5 oligomerization is itself reliant on Ca^{2+} ions as a cofactor, which is not considered in our model.

2.3 Analytical model for binding of multivalent probes in the presence of monovalent competitors

Following the procedure used to calculate partition functions for cofactors, we calculate the bound partition function in the presence of monovalent competitors. The difference is that binding of a competitor to a ligand inhibits formation of a bond with a receptor. We consider the effect of monovalent competitors at concentration c_{mc} (where we refer to unbound

competitors) that can bind to receptors with affinity $K_{\mathrm{d,R-mc}}$, or to ligands with affinity $K_{\mathrm{d,L-mc}}$. The partition sum over all bound states between a multivalent probe and surface receptors is

$$Q_{\mathrm{b}} = v_{\mathrm{b}} \sum_{i=1}^{\min[n_{\mathrm{L}},n_{\mathrm{R}}]} \Omega(i)\, e^{-i\Delta G/k_{\mathrm{B}}T} (v_{\mathrm{eff}}\rho_0)^{n_{\mathrm{L}}-i} \left(1+\frac{c_{\mathrm{mc}}}{K_{\mathrm{d,R-mc}}}\right)^{n_{\mathrm{R}}-i} \left(1+\frac{c_{\mathrm{mc}}}{K_{\mathrm{d,L-mc}}}\right)^{n_{\mathrm{L}}-i}, \quad \text{[S8A]}$$

where the binding of competitors to free ligands or receptors alone is treated in exactly the same way as binding of cofactors (Eq. [S1A]).

The corresponding partition function summing over all unbound states has the same form as the case of cofactors (Eq. [S1B]),

$$Q_{\mathrm{u}} = (\rho_0 N_{\mathrm{A}})^{-1} (v_{\mathrm{eff}}\rho_0)^{n_{\mathrm{L}}} \left(1+\frac{c_{\mathrm{mc}}}{K_{\mathrm{d,R-mc}}}\right)^{n_{\mathrm{R}}} \left(1+\frac{c_{\mathrm{mc}}}{K_{\mathrm{d,L-mc}}}\right)^{n_{\mathrm{L}}}. \quad \text{[S8B]}$$

The ratio of these two partition functions determines the interaction free energy,

$$e^{-F/k_{\mathrm{B}}T} = \frac{Q_{\mathrm{b}}}{Q_{\mathrm{u}}} = v_{\mathrm{b}}\rho_0 N_{\mathrm{A}} \sum_{i=1}^{\min[n_{\mathrm{L}},n_R]} \Omega(i) \left[\frac{e^{-\Delta G/k_{\mathrm{B}}T}}{v_{\mathrm{eff}}\rho_0\left(1+\frac{c_{\mathrm{mc}}}{K_{\mathrm{d,R-mc}}}\right)\left(1+\frac{c_{\mathrm{mc}}}{K_{\mathrm{d,L-mc}}}\right)}\right]^{i}. \quad \text{[S9]}$$

This equation is equivalent to the standard expression without monovalent competitors,[7, 16] i.e., $c_{\mathrm{mc}} = 0$, but with a rescaled interaction energy

$$e^{-\Delta G^{(\mathrm{mc})}/k_{\mathrm{B}}T} = e^{-\Delta G/k_{\mathrm{B}}T} \Big/ \left[\left(1+\frac{c_{\mathrm{mc}}}{K_{\mathrm{d,R-mc}}}\right)\left(1+\frac{c_{\mathrm{mc}}}{K_{\mathrm{d,L-mc}}}\right)\right]. \quad \text{[S10A]}$$

Equivalently, we can write the rescaling of affinity $K_{\mathrm{d}} = \rho_0 e^{\Delta G/k_{\mathrm{B}}T}$ as

$$K_{\mathrm{d}}^{(\mathrm{mc})} = K_{\mathrm{d}} \left(1+\frac{c_{\mathrm{mc}}}{K_{\mathrm{d,R-mc}}}\right)\left(1+\frac{c_{\mathrm{mc}}}{K_{\mathrm{d,L-mc}}}\right). \quad \text{[S10B]}$$

We stress that we did not need to assume a specific functional form for the number of states $\Omega(i)$, which is determined by the details of the multivalent interaction. Therefore, the rescaling (Eq. [S10A-B]) is generally applicable for any multivalent interaction. Since the free energy F determines all properties of the multivalent interaction, we conclude that monovalent competitors do not affect the nature of multivalent binding or superselectivity. Instead, the effect of monovalent competitors that bind to receptors and ligands can be fully captured by using a rescaled affinity constant (Eq. [S10B]).

In many practical cases the monovalent competitors bind to receptors but not to ligands, in which case the last term in Eq. [S10B] vanishes, $c_{\mathrm{mc}}/K_{\mathrm{d,L-mc}} = 0$, and thus $K_{\mathrm{d}}^{(\mathrm{mc})} = K_{\mathrm{d}}\left(1 + c_{\mathrm{mc}}/K_{\mathrm{d,R-mc}}\right)$, as given in Eq. [5] in the main text. Equivalently, if the competitors bind to ligands but not receptors, then $K_{\mathrm{d}}^{(\mathrm{mc})} = K_{\mathrm{d}}\left(1 + c_{\mathrm{mc}}/K_{\mathrm{d,L-mc}}\right)$. On the other hand, if more than one type of competitors is present in the system, they must simply be added to the rescaling obtaining

$$K_{\mathrm{d}}^{(\mathrm{mc})} = K_{\mathrm{d}} \left(1+\sum_{i=1}^{m}\frac{c_{\mathrm{mc},i}}{K_{\mathrm{d,R-mc},i}}\right)\left(1+\sum_{j=1}^{n}\frac{c_{\mathrm{mc},j}}{K_{\mathrm{d,L-mc},j}}\right), \quad \text{[S10C]}$$

where *m* and *n* are the total numbers of competitor types binding to receptors and ligands, respectively. The subscripts *i* and *j* denote the competitor types, $c_{\mathrm{mc,i}/j}$ their respective concentrations, and $K_{\mathrm{d,R-mc,i}}$ and $K_{\mathrm{d,L-mc},j}$ their corresponding affinity constants.

Multivalent interactions are typically superselective with respect to the affinity constant ($\alpha_{K_\mathrm{d}} = \mathrm{d}\log\Gamma_\mathrm{P}/\mathrm{d}\log K_\mathrm{d} > 1$). Since the concentration of monovalent competitors c_mc or cofactors c_cf modulates the effective affinity K_d (Eqs. [S3] and [S10B-C]), the multivalent interaction can be superselective with respect to c_mc ($\alpha_\mathrm{mc} = \mathrm{d}\log\Gamma_\mathrm{P}/\mathrm{d}\log c_\mathrm{mc} > 1$) or c_cf ($\alpha_\mathrm{cf} = \mathrm{d}\log\Gamma_\mathrm{P}/\mathrm{d}\log c_\mathrm{cf} > 1$), respectively.

2.4 HA binding to CD44-presenting cells – Estimating CD44 surface densities from the analysis of HA polysaccharide binding in competition with HA_8

Figure 3C shows example data for the effect of monovalent competitors for the case of HA binding to CD44 receptors. As described in the main text, a value of $n_\mathrm{R} = 0.03K_\mathrm{d}v_\mathrm{eff}$ was determined by fitting the experimental data with a simple analytical model (Eqs. [1-2] with the re-scaled affinity, Eq. [5]) and reasonable assumptions for all other parameters. The 'chemical' repeat unit of HA polysaccharides is a disaccharide, and the size ('footprint') of the HA binding site in CD44 is a decasaccharide. This entails a 5-fold degeneracy of the binding sites, and we therefore assume $K_\mathrm{d} = K_\mathrm{d,R-mc}/5 = 10$ µM for the interaction between the HA polysaccharide and CD44. This ensures that the binding of one receptor to the polymer is accurately represented, while the binding of multiple receptors to a polymer is reasonably approximated. We further take $v_\mathrm{eff} = a^3 N_\mathrm{A} e^{U_\mathrm{poly}/k_\mathrm{B}T}$, with $a = (4\pi/3)^{1/3} R_\mathrm{g}$ and $R_\mathrm{g} = 90$ nm for 1 MDa HA.[17] The energy U_poly (defined in Ref. 9) accounts for conformational entropy costs associated with confining the HA polymer to the cell surface and to the receptor binding sites. The exact magnitude of U_poly is unknown for native HA of the size considered here. In our previous work with modified HA chains,[9] we had estimated U_poly to be in the range of a few $k_\mathrm{B}T$, somewhat dependent on the polymer valency and linker type. If we conservatively estimate U_poly to lie between 0 and 5 $k_\mathrm{B}T$, then this results in n_R values between 0.55 and 82, corresponding to root-mean-square distances between receptors, $\sqrt{a^2/n_\mathrm{R}}$, ranging between approximately 16 and 200 nm. These values are broadly consistent with reported CD44 cell surface coverages,[18-21] indicating that our model makes reasonable quantitative predictions despite its simplicity.

3 SUPPORTING REFERENCES

1. Bérat, R.; Rémy-Zolghadry, M.; Gounou, C.; Manigand, C.; Tan, S.; Saltó, C.; Arenas, E.; Bordenave, L.; Brisson, A. R., Peptide-presenting two-dimensional protein matrix on supported lipid bilayers: An efficient platform for cell adhesion. *Biointerphases* **2007,** *2*, 165-172.
2. Bouter, A.; Gounou, C.; Berat, R.; Tan, S.; Gallois, B.; Granier, T.; d'Estaintot, B. L.; Poschl, E.; Brachvogel, B.; Brisson, A. R., Annexin-A5 assembled into two-dimensional arrays promotes cell membrane repair. *Nat Commun* **2011,** *2*, 270.
3. Richter, R. P.; Lai Kee Him, J.; Tessier, B.; Tessier, C.; Brisson, A., On the kinetics of adsorption and two-dimensional self-assembly of annexin A5 on supported lipid bilayers. *Biophys J* **2005,** *89*, 3372-3385.
4. Carton, I.; Brisson, A. R.; Richter, R. P., Label-free detection of clustering of membrane-bound proteins. *Anal Chem* **2010,** *82*, 9275-9281.
5. Reviakine, I.; Johannsmann, D.; Richter, R. P., Hearing what you cannot see and visualizing what you hear: interpreting quartz crystal microbalance data from solvated interfaces. *Anal Chem* **2011,** *83*, 8838-8848.
6. Richter, R. P.; Brisson, A., QCM-D on mica for parallel QCM-D - AFM studies. *Langmuir* **2004,** *20*, 4609-4613.

7. Curk, T.; Dobnikar, J.; Frenkel, D., Design principles for super selectivity using multivalent interactions. In *Multivalency: Concepts, Research & Applications*, Haag, R.; Huskens, J.; Prins, L.; Ravoo, B. J., Eds. John Wiley & Sons: Oxford, 2018.
8. Curk, T.; Dobnikar, J.; Frenkel, D., Optimal multivalent targeting of membranes with many distinct receptors. *Proc Natl Acad Sci U S A* **2017,** *114*, 7210-7215.
9. Dubacheva, G. V.; Curk, T.; Auzely-Velty, R.; Frenkel, D.; Richter, R. P., Designing multivalent probes for tunable superselective targeting. *Proc Natl Acad Sci U S A* **2015,** *112*, 5579-84.
10. Jeppesen, B.; Smith, C.; Gibson, D. F.; Tait, J. F., Entropic and enthalpic contributions to annexin V-membrane binding: a comprehensive quantitative model. *J Biol Chem* **2008,** *283*, 6126-35.
11. Dubacheva, G. V.; Curk, T.; Frenkel, D.; Richter, R. P., Multivalent recognition at fluid surfaces: The interplay of receptor clustering and superselectivity. *J Am Chem Soc* **2019,** *141*, 2577-2588.
12. Arraud, N. Kinetics of Annexin-A5 binding to model membranes studied by flow cytometry and development of a new method for quantifying plasmatic microparticles. PhD thesis, Bordeaux-1 University, 2011.
13. Cezanne, L.; Lopez, A.; Loste, F.; Parnaud, G.; Saurel, O.; Demange, P.; Tocanne, J. F., Organization and dynamics of the proteolipid complexes formed by annexin V and lipids in planar supported lipid bilayers. *Biochemistry* **1999,** *38*, 2779-86.
14. Jin, M.; Smith, C.; Hsieh, H. Y.; Gibson, D. F.; Tait, J. F., Essential role of B-helix calcium binding sites in annexin V-membrane binding. *J Biol Chem* **2004,** *279*, 40351-7.
15. Tait, J. F.; Gibson, D. F.; Smith, C., Measurement of the affinity and cooperativity of annexin V-membrane binding under conditions of low membrane occupancy. *Anal Biochem* **2004,** *329*, 112-9.
16. Martinez-Veracoechea, F. J.; Frenkel, D., Designing super selectivity in multivalent nano-particle binding. *Proc Natl Acad Sci U S A* **2011,** *108*, 10963-8.
17. Takahashi, R.; Al-Assaf, S.; Williams, P. A.; Kubota, K.; Okamoto, A.; Nishinari, K., Asymmetrical-flow field-flow fractionation with on-line multiangle light scattering detection. 1. Application to wormlike chain analysis of weakly stiff polymer chains. *Biomacromolecules* **2003,** *4*, 404-409.
18. Visintin, A.; Mazzoni, A.; Spitzer, J. H.; Wyllie, D. H.; Dower, S. K.; Segal, D. M., Regulation of Toll-like receptors in human monocytes and dendritic cells. *J. Immunol.* **2001,** *166*, 249-55.
19. Alves, C. S.; Burdick, M. M.; Thomas, S. N.; Pawar, P.; Konstantopoulos, K., The dual role of CD44 as a functional P-selectin ligand and fibrin receptor in colon carcinoma cell adhesion. *Am. J. Physiol. Cell Physiol.* **2008,** *294*, C907-916.
20. Wolny, P. M.; Banerji, S.; Gounou, C.; Brisson, A. R.; Day, A. J.; Jackson, D. G.; Richter, R. P., Analysis of CD44-hyaluronan interactions in an artificial membrane system: insights into the distinct binding properties of high and low molecular weight hyaluronan. *J Biol Chem* **2010,** *285*, 30170-30180.
21. Freeman, S. A.; Vega, A.; Riedl, M.; Collins, R. F.; Ostrowski, P. P.; Woods, E. C.; Bertozzi, C. R.; Tammi, M. I.; Lidke, D. S.; Johnson, P.; Mayor, S.; Jaqaman, K.; Grinstein, S., Transmembrane pickets connect syto- and pericellular skeletons forming barriers to receptor engagement. *Cell* **2018,** *172*, 305-317 e10.
